# Boundary cap neural crest stem cells homotopically implanted to the injured dorsal root transitional zone give rise to different types of neurons and glia in adult rodents

**DOI:** 10.1186/1471-2202-15-60

**Published:** 2014-05-05

**Authors:** Carl Trolle, Niclas Konig, Ninnie Abrahamsson, Svitlana Vasylovska, Elena N Kozlova

**Affiliations:** 1 Department of Neuroscience, Uppsala University Biomedical Center, Box 593, SE-751 24 Uppsala, Sweden

**Keywords:** Neural stem cell, Sensory neuron, Spinal cord injury, Cell differentiation, Nerve regeneration, Cell replacement

## Abstract

**Background:**

The boundary cap is a transient group of neural crest-derived cells located at the presumptive dorsal root transitional zone (DRTZ) when sensory axons enter the spinal cord during development. Later, these cells migrate to dorsal root ganglia and differentiate into subtypes of sensory neurons and glia. After birth when the DRTZ is established, sensory axons are no longer able to enter the spinal cord. Here we explored the fate of mouse boundary cap neural crest stem cells (bNCSCs) implanted to the injured DRTZ after dorsal root avulsion for their potential to assist sensory axon regeneration.

**Results:**

Grafted cells showed extensive survival and differentiation after transplantation to the avulsed DRTZ. Transplanted cells located outside the spinal cord organized elongated tubes of Sox2/GFAP expressing cells closely associated with regenerating sensory axons or appeared as small clusters on the surface of the spinal cord. Other cells, migrating into the host spinal cord as single cells, differentiated to spinal cord neurons with different neurotransmitter characteristics, extensive fiber organization, and in some cases surrounded by glutamatergic terminal-like profiles.

**Conclusions:**

These findings demonstrate that bNCSCs implanted at the site of dorsal root avulsion injury display remarkable differentiation plasticity inside the spinal cord and in the peripheral compartment where they organize tubes associated with regenerating sensory fibers. These properties offer a basis for exploring the ability of bNCSCs to assist regeneration of sensory axons into the spinal cord and replace lost neurons in the injured spinal cord.

## Background

Boundary cap neural crest stem cells (bNCSCs) are neural crest derivatives that populate the entry/exit points of spinal roots during embryonic development [1]. They appear to participate in regulating growth of sensory axons into the spinal cord [2], and prevent spinal motor neurons [3] and central neuroglial cells [4] to enter the peripheral nervous system (PNS) [2,5,6]. In later developmental stages a morphologically well-defined glial interface appears in the dorsal roots at their junctions with the spinal cord, the dorsal root transitional zone (DRTZ). This forms the boundary between the peripheral and central nervous system and is characterized by peripherally extending projections of CNS tissue, rich in astroglial processes, which interdigitate with the PNS tissue compartment [7,8]. The DRTZ is an area of fundamental importance for axon regeneration in the spinal cord. At some point during development, which in the rat occurs a few days after birth [9], the DRTZ becomes impenetrable for regenerating axons. Thus, injured dorsal root axons which regenerate in the peripheral compartment of the dorsal root, are unable to enter the spinal cord [10]. Since boundary cap cells are part of the growth permissive environment at the dorsal root-spinal cord junction during development we therefore tested whether bNCSCs placed to their native position can assist sensory axon regeneration after dorsal root avulsion injury [11].

bNCSCs are also a source of neural crest derived stem cells that give rise to Schwann cells of spinal roots and constitute the third wave of cell migration to dorsal root ganglia, where they give rise to nociceptive and thermoceptive neurons [12,13], as well as to satellite cells [12]. bNCSCs are able to generate mature Schwann cells *in vitro* and after transplantation to the adult sciatic nerve [14], and can be genetically driven to generate subtype specific sensory neurons after transplantation to the dorsal root ganglion cavity of adult mice [15]. Furthermore, bNCSCs are able to generate central glial and neuronal cells *in vitro* and after transplantation *in vivo*[16,17]. Based on these observations we also examined the migratory properties and phenotypic differentiation of bNCSCs in the peripheral and central compartments after implantation at their homotypic site, the interface between the central and peripheral nervous system in the dorsal root avulsion injury model.

## Methods

### Animals

Recipients for transplantation were adult female Sprague–Dawley rats (n = 6; 250–280 g; Mollegaard, Denmark) and adult male nu/nu NMRI mice (n = 13; 25–30 g; Mollegaard, Denmark). All animal experiments were approved by the Local Ethical Committee for Animal Experimentation, Uppsala, as required by Swedish Legislation and in accordance with European Union Directives.

### Culture

Primary cultures of bNCSCs (NL-38) from passage 20 were prepared according to previously published protocol [13,15]. Briefly, the dorsal root ganglia (DRGs), along with the dorsal and ventral roots, were mechanically separated from the isolated spinal cord of heterozygous 11-days embryos from C57BL/6- β-actin enhanced green fluorescent protein (eGFP) transgenic mice (Jackson Laboratories, Bar Harbor, Maine, USA) and enzymatically dissociated using Collagenase/Dispase (1 mg/ml) and DNase (0.5 mg/ml) for 30 minutes at room temperature. Cells were plated at 0.5–1 X 10^5^ cells/cm^2^ in N2 medium containing B27 (Gibco, Grand Island, NY, http://www.invitrogen.com) as well as epidermal growth factor (PeproTech, Rocky Hill, New Jersey, USA, 20 ng/ml), and basic fibroblast growth factor (bFGF; R&D Systems, Minneapolis, http://www.rndsystems.com, 20 ng/ml). After 12 hours, non-adherent cells were removed together with half of the medium before adding fresh medium. The medium was changed every other day until neurospheres were observed after approximately two weeks of culture. Neurospheres were then kept free-floating in propagation medium (PROP: DMEM/F-12 medium (Invitrogen, n° 31330–038) supplemented with B27 (Invitrogen, n° 17504–044) and N2 (Invitrogen, n° 17502–048) and containing 20 ng/ml bFGF (Invitrogen, n° 13256–029) and 20 ng/ml EGF (R&D system, n° 236-EG). Since the bNCSCs were prepared from C57BL/6- β-actin enhanced green fluorescent protein (eGFP) transgenic mice, all cells in the generated neurospheres, which were used for transplantation, expressed GFP and were easily visualized after transplantation.

### Surgery

Six adult rats and 7 adult nude mice were subjected to dorsal root avulsion with subsequent transplantation and 6 mice were subjected to transplantation without avulsion. Animals were anesthetized with a mixture of ketamine, xylazine and acepromazine (at 100, 20, and 3 μg/g bodyweight respectively) intraperitoneally and the left L3-L6 (rats) or L3-L5 (mice) dorsal roots were exposed via a partial laminectomy and durectomy, and bNCSCs were placed on the top of uninjured DRTZ L3-L6, or on the top of pulled and re-attached dorsal roots on the surface of the spinal cord [11]. The wound was closed in layers and the rats were maintained on immunosuppression with Cyclosporine A (Sandimmun®, Novartis) during their postoperative survival period whereas nude mice did not receive Cyclosporine.

### Immunohistochemistry

After one to two weeks (n = 10) and one month (n = 9) recipient animals were re-anesthetized and perfused with warm saline (~38°C) followed by a fixative solution containing ice cold 4% formaldehyde (w/v) and 14% saturated picric acid (v/v) in phosphate buffered saline (PBS) (~4°C; pH 7.35-7.45). The left L3-L6 spinal cord segments with attached dorsal roots were removed, post-fixed at 4°C for four hours, and cryoprotected overnight in PBS containing 15% sucrose. Serial transverse sections (14 μm) were cut through the spinal cord on a cryostat, and placed on SuperFrost® Plus glass slides (Menzel-Gläser, Braunschweig, Germany). Sections were pre-incubated with blocking solution (1% bovine serum albumin, 0.3% Triton X-100 and 0.1% NaN_3_ in PBS) for one hour at room temperature and then incubated overnight at 4°C with primary antibodies. After washing with PBS, appropriate secondary antibodies were applied for 4 h at room temperature (Table 1).

**Table 1:** Antibodies used for immunohistochemistry

***Primary***					***Secondary***				
**Antigen**	**Host**	**Cat no.**	**Source**	**Dilution**	**Antibody**	**Target**	**Cat no.**	**Source**	**Dilution**
β-tubulin	Mouse	32-2600	Invitrogen	1:500	Alexa 350	Mouse	A10035	Invitrogen	1:1000
Calbindin	Rabbit	CB-38a	Swant	1:5000	Alexa 488	Mouse	A21202	Invitrogen	1:1000
CGRP	Goat	ab36001	Abcam	1:200	Alexa 488	Rabbit	A11008	Invitrogen	1:1000
ChAT	Goat	AB144P	Millipore	1:100	Alexa 546	Chicken	A11040	Invitrogen	1:1000
cJun	Mouse	610326	BD Transduction Labs	1:250	Alexa 555	Goat	A21432	Invitrogen	1:1000
CNPase	Mouse	SMI 91R	Covance	1:1000	Alexa 555	Rabbit	A31572	Invitrogen	1:1000
Doublecortin	Rabbit	ab18723	Abcam	1:1000	Alexa 594	Mouse	A11032	Invitrogen	1:1000
GFAP	Rabbit	2016-04	DAKO	1:500	Cy3	Guinea pig	706-165-148	Jackson	1:500
HuC/D	Mouse	A21271	Invitrogen	1:200					
Map2	Chicken	ab5392	Abcam	1:1500					
Olig2	Goat	Sc-19967	Santa Cruz	1:100					
p75	Rabbit	AB1554	Millipore	1:200					
Ret	Goat	AF482	R&D Systems	1:20					
RT97	Mouse	1178709	Mannheim boehringer	1:50					
Sox2	Goat	Sc-17320	Santa Cruz	1:200					
TrkA	Rabbit	06-574	Millipore	1:500					
TrkB	Goat	AF1494	R&D Systems	1:500					
TrkC	Goat	AF1404	R&D Systems	1:500					
VGluT2	Guinea pig	AB2251	Millipore	1:5000					

### Microscopy

Immunolabeled sections were analyzed under a Nikon Eclipse E800 fluorescence microscope and for photography, a Nikon DXM1200F digital camera system was used. Fluorescent sections were also analyzed by confocal microscopy using a Zeiss LSM 510 META system (Oberkochen, Germany). Captured images were auto-leveled using Adobe Photoshop software. To study the interrelations between transplanted cells and host tissues, photos were acquired with a Zeiss LSM 510 Meta confocal microscope and 63x/1.4 NA plan-Apochromate lens using a laser line of 561 nm and LP 565 emission filter. Z-stacks were acquired with an optical slice thickness of 0.8 μm and an interval of 0.5 μm.

### Cell counts in the peripheral and central compartments

The total number of Hoechst/eGFP-positive cells and the number of Hoechst/eGFP-positive cells labeled with Sox2 and GFAP in one month transplants were counted on confocal images from every 10^th^ slide in 3 animals. For calculating the proportion of GFAP labeled cells 150–300 Hoechst/eGFP-positive cells were analyzed per slide. For the Sox2 100–200 cells were counted per slide using confocal images.

## Results and discussion

**Figure 1:**
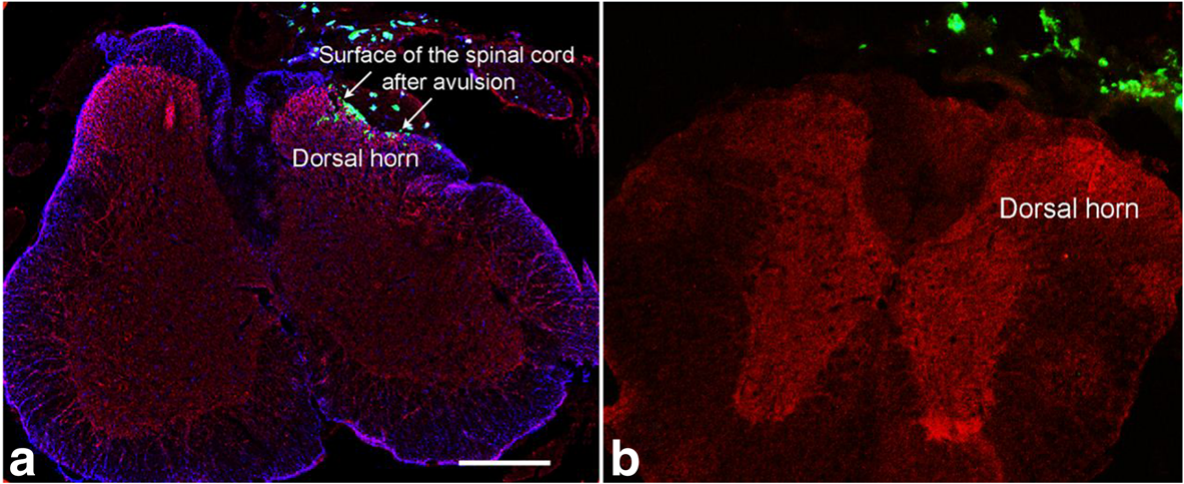
**Transverse section through the mouse spinal cord two weeks after transplantation of bNCSCs to the avulsed lumbar spinal cord (a) and on the surface of non-avulsed spinal cord (b).** eGFP-positive bNCSCs are located in the peripheral compartment of the dorsal root as well as within the host spinal cord **(a)** but only on the surface when dorsal roots were not avulsed **(b)**. Blue, GFAP, red, microtubule-associated protein (MAP)2. **b**. Transplanted NCSCs are green (eGFP). Scale bar = 400 μm.

After transplantation of bNCSCs to the site of avulsed and re-attached dorsal roots, numerous eGFP-positive cells were observed in two weeks and throughout the experimental period (one month) in the proximal part of the avulsed dorsal roots, along the dorsal and dorso-lateral surface of the spinal cord, as well as within the superficial part of the ipsilateral dorsal horn (Figure 1a). In contrast, bNCSCs placed on the dorsal surface of the L3-6 spinal cord in animals with intact dorsal roots showed complete absence of migration into the spinal cord (Figure 1b). Thus, migration of bNCSCs into the spinal cord is likely to be the result of the combined production and release of growth/migration supporting factors by cells in the peripheral [18], and central nervous system that respond to the degenerative processes in the spinal cord [19] or at the DRTZ [20].

**Figure 2:**
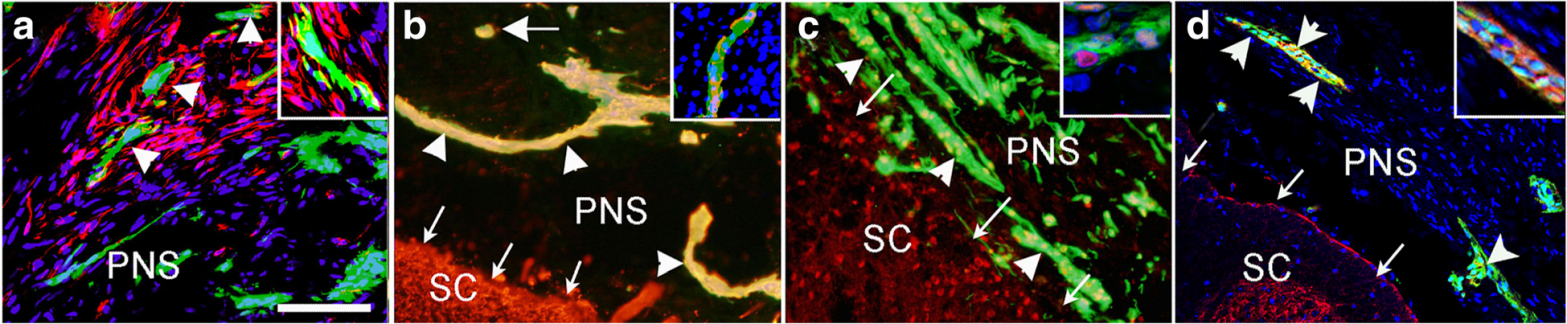
**Transverse sections through the spinal cord with adjacent transplant.** bNCSCs (green) located outside the spinal cord do not express the Schwann cell marker p75 (**a**; red p75, eGFP bNCSCs, blue Hoechst), are arranged in elongated tubes (arrowheads) and express glial markers GFAP (**b**, red), and Sox2 (**c**, red), or as clusters or single cells and express TrkB (**d**, red). Inserts a-c are merged images of eGFP-positive cells, the respective antibody labeling and Hoechst labeling. Insert in d shows higher magnification of TrkB expressing bNCSCs. Scale bar in **a**-**d** = 50 μm; inserts = confocal images showing close association of bNCSC with Schwann cells **(a)**, GFAP-expressing bNCSCs in the tubes **(b)**, Sox2-expressing bNCSCs in tubes **(c)**, TrkB-expressing bNCSCs in the PNS compartment **(d)**.

We noted striking differences in the differentiation of bNCSCs located in the PNS outside the spinal cord compared to cells, which had migrated into the spinal cord. Thus cells located outside the spinal cord were negative for doublecortin (DCX), a marker of migrating neurons [21], whereas cells inside the spinal cord did express DCX (Additional file 1: Figure S1), thereby resembling previously described migrating bNCSCs after transplantation in a spinal cord demyelinating model [16]. Some bNCSCs outside the spinal cord expressed nestin whereas all bNCSCs inside the spinal cord were nestin-negative (Additional file 2: Figure S2). bNCSCs were closely associated with p75-expressing Schwann cells (Figure 2a) and some bNCSCs in the PNS compartment expressed p75 (Additional file 3: Figure S3).

In all cases, bNCSCs in the peripheral compartment of the dorsal root displayed extensive formation of tubular-like structures and clusters of cells on the surface of the spinal cord two weeks and one month after transplantation (Figure 2b). In contrast, eGFP-positive cells that had migrated into the spinal cord appeared only as single cells, sometimes forming small clusters (Additional file 4: Figure S4). Counts showed that the majority of eGFP-positive cells (around 75%) in the tubes were positive for GFAP (Figure 2b) and Sox2 (around 90%) (Figure 2c). Some peripherally located bNCSCs expressed the neurotrophin receptor TrkB (Figure 2d), but were negative for TrkA and TrkC (not shown), as previously described for boundary cap cells [22]. The combined expression of Sox2 and GFAP was recently described in a subpopulation of astrocytes that appear to play a crucial role in organizing glial scar after spinal cord injury [23]. Some of the tube-forming Sox2 expressing bNCSCs were positive for the transcription factor cJun resembling Schwann cells in the tracks that assist axonal regeneration [24,25]. This suggest that some of the transplanted bNCSC may share properties similar to the Schwann-repair cell (or Bungner cell [25]) or that they are able to respond to nerve injuries in a similar fashion.

**Figure 3:**
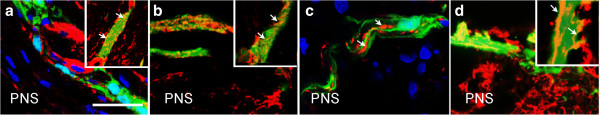
**Transverse sections through the spinal cord with adjacent transplant.** Elongated tubes formed by bNCSCs (green) outside the spinal cord are associated with regenerating fibers (red) from different subpopulations of sensory neurons: IB4 **(a)**, /RET **(b)**, CGRP **(c)**, RT97 **(d)**. Note IB4 labeling of macrophages **(a)**. Due to their undulating course, sensory axons appear in the thin sections as short segments (arrows) associated with eGFP-positive cells. Inserts **a**, **b** and **d** show details of the relationship between bNCSC forming tubes and the respective sensory axon subtypes. Scale bar: **a** = 50 μm, **b** = 100 μm, **c** = 25 μm, **d** = 100 μm; inserts: **a** = 50 μm, **b** = 50 μm, **c** = 25 μm, **d** = 50 μm;.

To determine the relationship between tube forming bNCSCs and dorsal root axons, we labeled subpopulations of sensory axons for *Bandeiraea simplicifolia* isolectin-B4 (IB4) or the tyrosine kinase receptor RET (non-myelinated, non-peptidergic; Figure 3a,b), calcitonin gene-related peptide (CGRP) (non-myelinated, peptidergic; Figure 3c) and with antibody RT97 (myelinated; Figure 3d). Whereas regenerating fibers were associated with bNCSC tubes outside the spinal cord, bNCSCs inside the spinal cord appeared as single cells and had no association with sensory fibers (Additional file 5: Figure S6). All subtypes of sensory fibers were present along eGFP-positive tubes, indicating that Sox2/GFAP-positive bNCSCs are able to provide a supporting terrain for growth of injured sensory axons. Proteoglycans produced by astrocytes in response to dorsal root injury are generally viewed as factors that prevent injured sensory axons from re-entering the adult spinal cord [26]. More recent studies have provided evidence that growing sensory axons are not repelled when they reach the DRTZ, but cease to grow after making synapse-like contacts with astrocytes within the spinal cord [27]. The GFAP-positive cells in the tubes thus appear to lack factors that induce stable contacts between growing sensory axons and bNCSC associated astrocytes.

**Figure 4:**
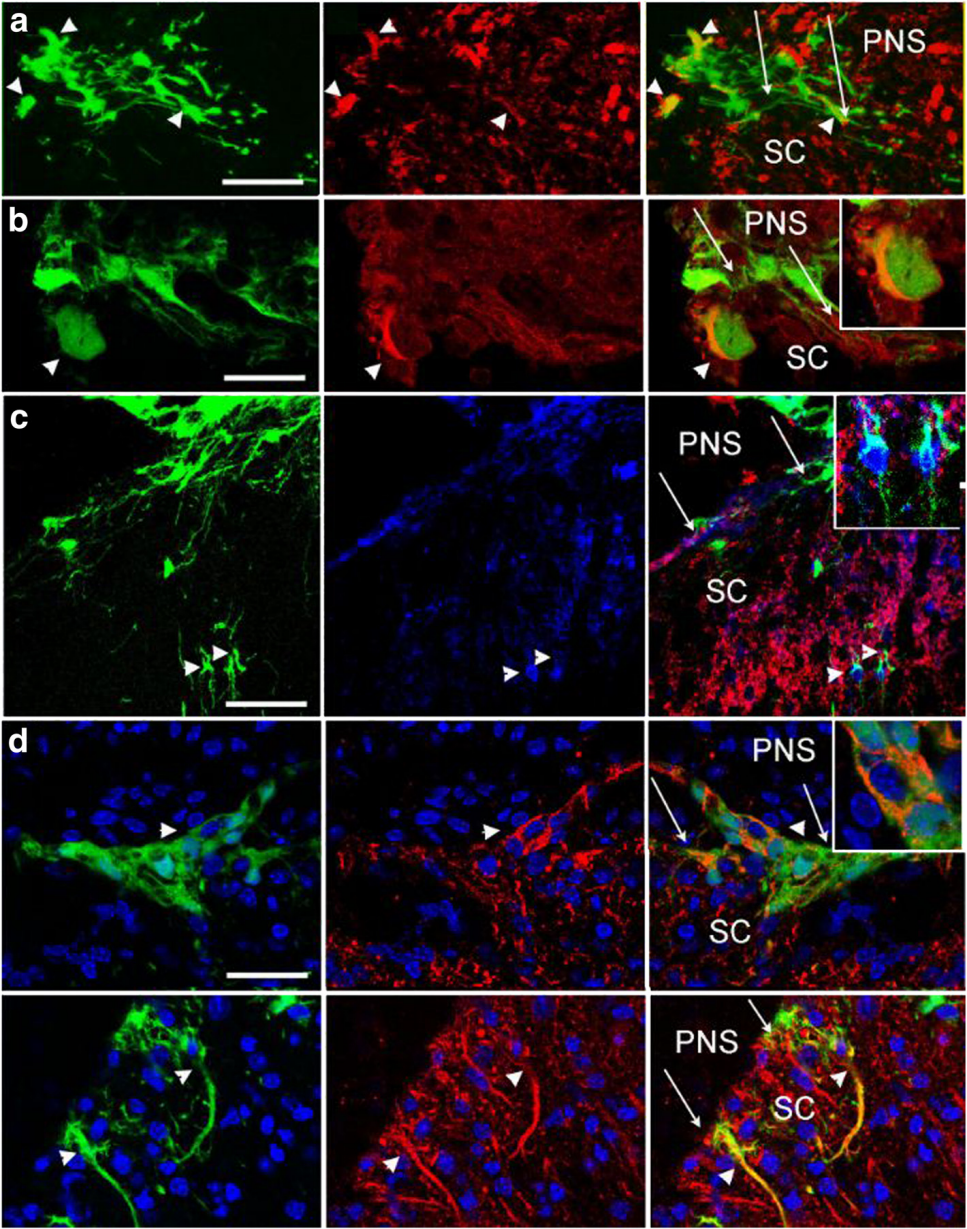
**Transverse sections trough the spinal cord with adjacent transplant.** bNCSCs (green) that have migrated into the host spinal cord express neuronal markers HuC/D (**a**, red), RET (**b**, red), Calbindin (**c**, blue), MAP2 (**c**, red), ChAT (**d**, 2 panels, red). Arrows show the surface of the spinal cord. Scale bar: **a** = 50 μm, **b** = 25 μm, **c** = 50 μm, **d**-**e** = 25 μm.

Only occasional transplanted eGFP-positive cells that had migrated into the host spinal cord expressed markers for central glia; GFAP (astrocytes; Additional file 4: Figure S4a) and CNPase (mature oligodendrocytes; Additional file 4: Figure S4b), and Olig2 (immature oligodendrocytes) was not detected (not shown). Some bNCSCs migrating into the spinal cord were positive for the RNA-binding protein HuC/D, a pan-neuronal marker [28] (Figure 4a), some expressed RET (Figure 4b), the calcium-binding protein Calbindin (Figure 4c), MAP2, or the cholinergic neuron marker choline acetyl transferase (ChAT) (Figure 4d). Labeling for VGluT2 showed VGluT2-positive profiles in close proximity of eGFP-positive cells that had migrated into the spinal cord (Additional file 6: Figure S5).

Thus, in contrast to glial or immature properties by bNCSCs in the PNS compartment, transplanted cells which had migrated into the spinal cord preferentially differentiated to neuronal, but not glial phenotypes. A previous study demonstrated extensive migration and differentiation to neuronal and glial phenotypes of bNCSCs transplanted into the subventricular zone (SVZ) of newborn mice [17]. bNCSCs transplanted to the demyelinated spinal cord of adult nude mice were found to generate myelinating Schwann cells and oligodendrocytes [16], and bNCSCs transplanted into the forebrain of newborn dysmyelinating mice (*Shiverer* mice) gave rise to central glia as well as to neurons [29]. The limated glial differentiation in the spinal cord in our study may be explained by the different environments in the immature SVZ and in conditions with pure de-/dysmyelination compared to the combination of axonal as well as myelin disintegration that occurs after dorsal root avulsion. Dorsal root avulsion also results in degeneration of second order neurons [30], extensive microglial, astroglial and vascular changes [31], processes which may provide additional stimuli for differentiation of bNCSCs towards neuronal phenotypes.

Neuronal differentiation of bNCSCs in the spinal cord occurred along several subtype lineages, including neurons expressing calbindin, ChAT or RET. Calbindin is expressed in both excitatory and inhibitory segmental and intersegmental interneurons [32]. ChAT-positive neurons are sparsely distributed but form a widely distributed network of fibers, which are proposed to modulate sensory transmission in the dorsal horn [33]. Neurons expressing the tyrosine kinase receptor RET include interneurons as well as neurons in lamina I giving rise to the spinothalamic tract [34]. These observations are evidence of a broad neuronal differentiation potential of bNCSCs that enter the spinal cord after transplantation. The observation of VGluT2 expressing profiles in immediate proximity of eGFP-positive cells suggest that these cells are contacted by host glutamatergic neurons.

## Conclusions

Taken together, bNCSCs transplanted to the site of dorsal root avulsion display good survival and remarkable plasticity by forming elongated and apparently growth permissive tubes in the peripheral compartment of the dorsal root, and by generating a variety of neuronal phenotypes after single cell migration into the host dorsal horn. These findings highlight the potential benefits of exploiting the properties of bNCSCs for neural repair.

## Competing interest

On behalf of the authors of the manuscript, I wish to state that the authors declare no financial or non-financial competing interests (EK).

## Authors’ contributions

CT carried out the transplantations, preparation of material, immunohistochemistry and dataanalysis. NA carried out confocal microscopy, preparation of material and immunohistochemistry. NK carried out surgery, preparation of material and data analysis. SV performed bNCSC cultures and confocal microscopy. EK conceived the study, and participated in its design and coordination and helped to write the manuscript. All authors contributed to writing and editing of drafts, and approved the final manuscript.

## Supplementary Material

Additional file 1: Figure S1 Transverse section through the spinal cord with adjacent transplants. Doublecortin (DCX) labeling (red). bNCSCs (green) located outside the spinal cord (SC) do not express DCX (a), whereas some bNCSCs that have migrated into the spinal cord are DCX-positive (b). Scale bar = 50 μm. Click here for file

Additional file 2: Figure S2 Transverse section through the spinal cord with adjacent transplants. Nestin labeling (red). Nestin is expressed in some bNCSCs (green) forming peripherally located tubes (a, arrows) but is absent in bNSCs that have migrated into the spinal cord (b). Scale bar: a = 50 μm, b = 10 μm. Click here for file

Additional file 3: Figure S3 Transverse section trough the spinal cord with adjacent two week eGFP-bNCSC transplant. Some of the cells in the PNS located tubes express the Schwann cell marker p75 (arrows). (p75-red; eGFP-bNCSCs). Scale bar = 25 μm. Click here for file

Additional file 4: Figure S4 bNCSCs located outside the spinal cord and on its surface express GFAP (red, arrowheads), whereas bNCSCs that have migrated into the spinal cord are GFAP-negative (a, arrows). Occasional cells inside the spinal cord express the oligodendroglial marker CNPase (b; red, arrows) Scale bar: a = 50 μm, b =10 μm. Click here for file

Additional file 5: Figure S6 bNCSCs (green) in were located outside and inside spinal cord and peripherally located tubes are associated with IB4 (a, red), RT97 (b, red) and CGRP (c, red)-expressing sensory axons (arrowheads), whereas bNCSCs inside the spinal cord do not display these associations (arrows). Scale bar: a = 200 μm, b = 100 μm, c = 200 μm. Click here for file

Additional file 6: Figure S5 bNCSCs (green) within the spinal cord are closely associated with VGlut2, a marker of glutamatergic terminals (red, arrows; blue - Hoechst). Scale bar = 10 μm. Click here for file
