# Supplementary figures and images for: Boundary cap neural crest stem cells homotopically implanted to the injured dorsal root transitional zone give rise to different types of neurons and glia in adult rodents
Source: BMC Neurosci. 2014 May 5;15:60. doi: 10.1186/1471-2202-15-60 (PMC4055944; doi:10.1186/1471-2202-15-60)

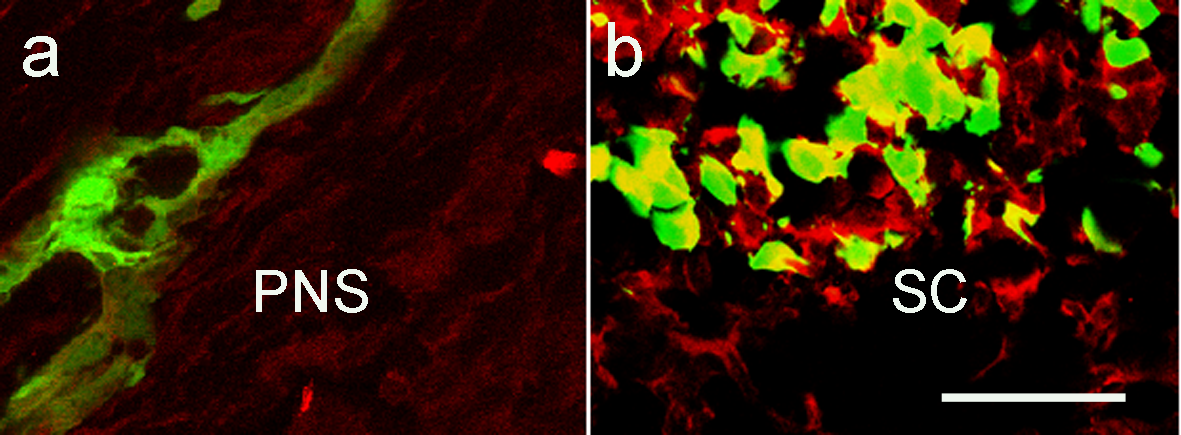

Supplement: Additional file 1: Figure S1 — Transverse section through the spinal cord with adjacent transplants. Doublecortin (DCX) labeling (red). bNCSCs (green) located outside the spinal cord (SC) do not express DCX (a), whereas some bNCSCs that have migrated into the spinal cord are DCX-positive (b). Scale bar = 50 μm. [file 1471-2202-15-60-S1.tiff]

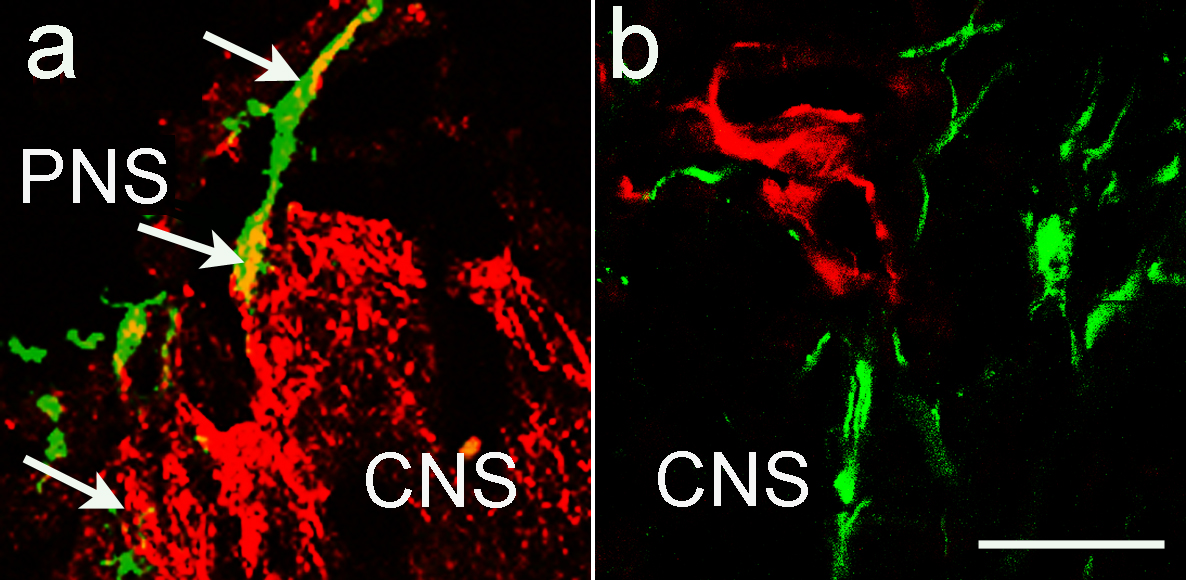

Supplement: Additional file 2: Figure S2 — Transverse section through the spinal cord with adjacent transplants. Nestin labeling (red). Nestin is expressed in some bNCSCs (green) forming peripherally located tubes (a, arrows) but is absent in bNSCs that have migrated into the spinal cord (b). Scale bar: a = 50 μm, b = 10 μm. [file 1471-2202-15-60-S2.tiff]

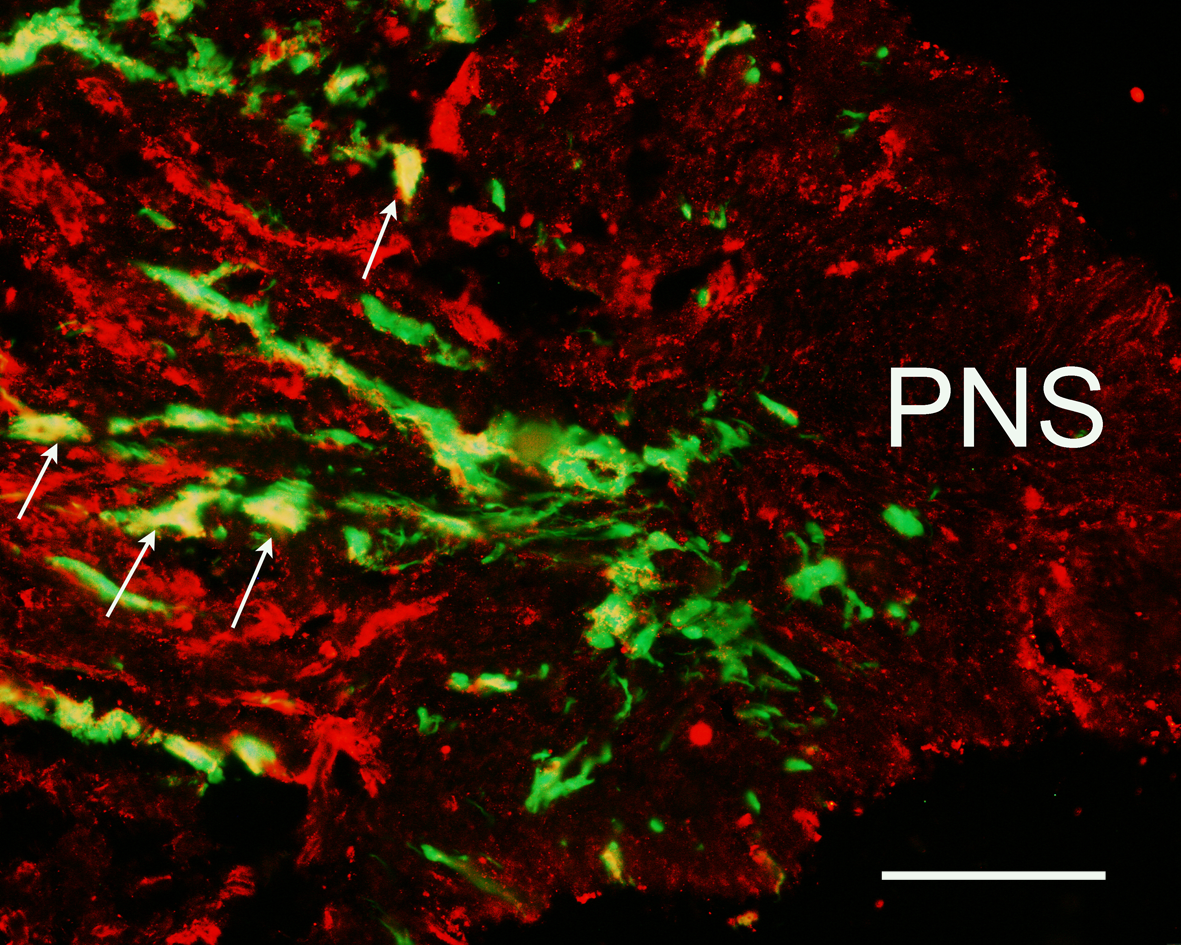

Supplement: Additional file 3: Figure S3 — Transverse section trough the spinal cord with adjacent two week eGFP-bNCSC transplant. Some of the cells in the PNS located tubes express the Schwann cell marker p75 (arrows). (p75-red; eGFP-bNCSCs). Scale bar = 25 μm. [file 1471-2202-15-60-S3.tiff]

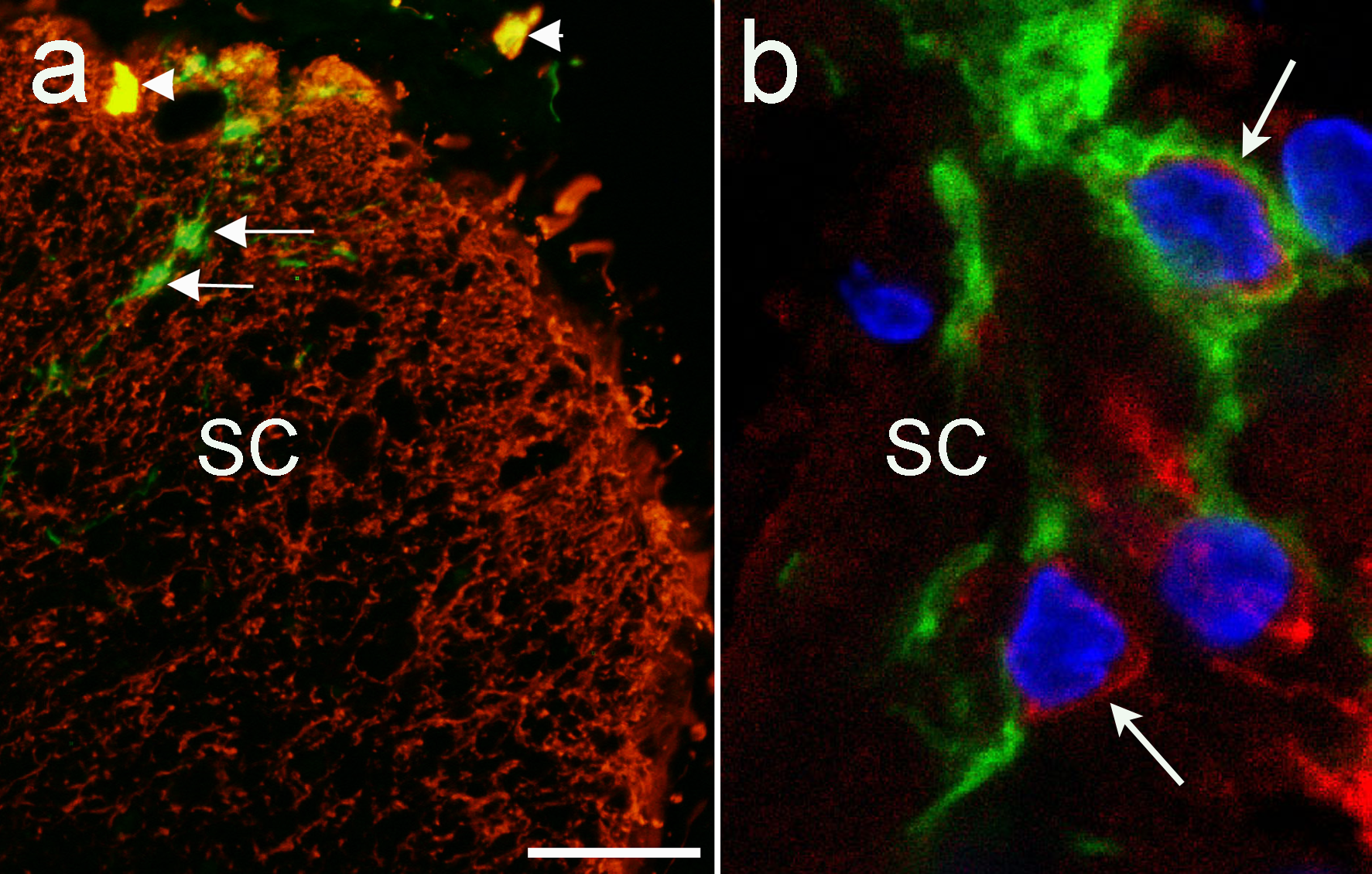

Supplement: Additional file 4: Figure S4 — bNCSCs located outside the spinal cord and on its surface express GFAP (red, arrowheads), whereas bNCSCs that have migrated into the spinal cord are GFAP-negative (a, arrows). Occasional cells inside the spinal cord express the oligodendroglial marker CNPase (b; red, arrows) Scale bar: a = 50 μm, b =10 μm. [file 1471-2202-15-60-S4.tiff]

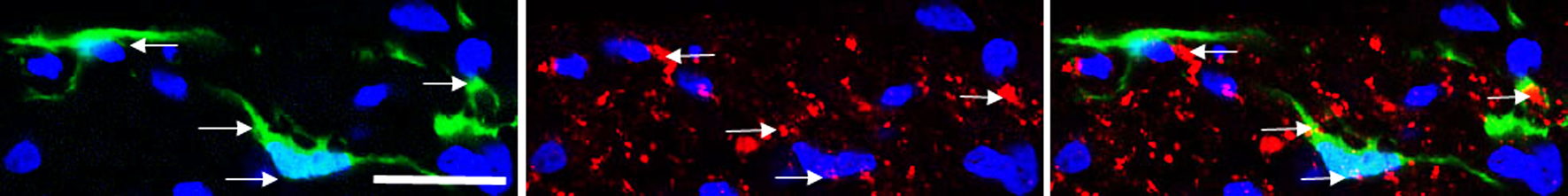

Supplement: Additional file 5: Figure S6 — bNCSCs (green) in were located outside and inside spinal cord and peripherally located tubes are associated with IB4 (a, red), RT97 (b, red) and CGRP (c, red)-expressing sensory axons (arrowheads), whereas bNCSCs inside the spinal cord do not display these associations (arrows). Scale bar: a = 200 μm, b = 100 μm, c = 200 μm. [file 1471-2202-15-60-S5.tiff]

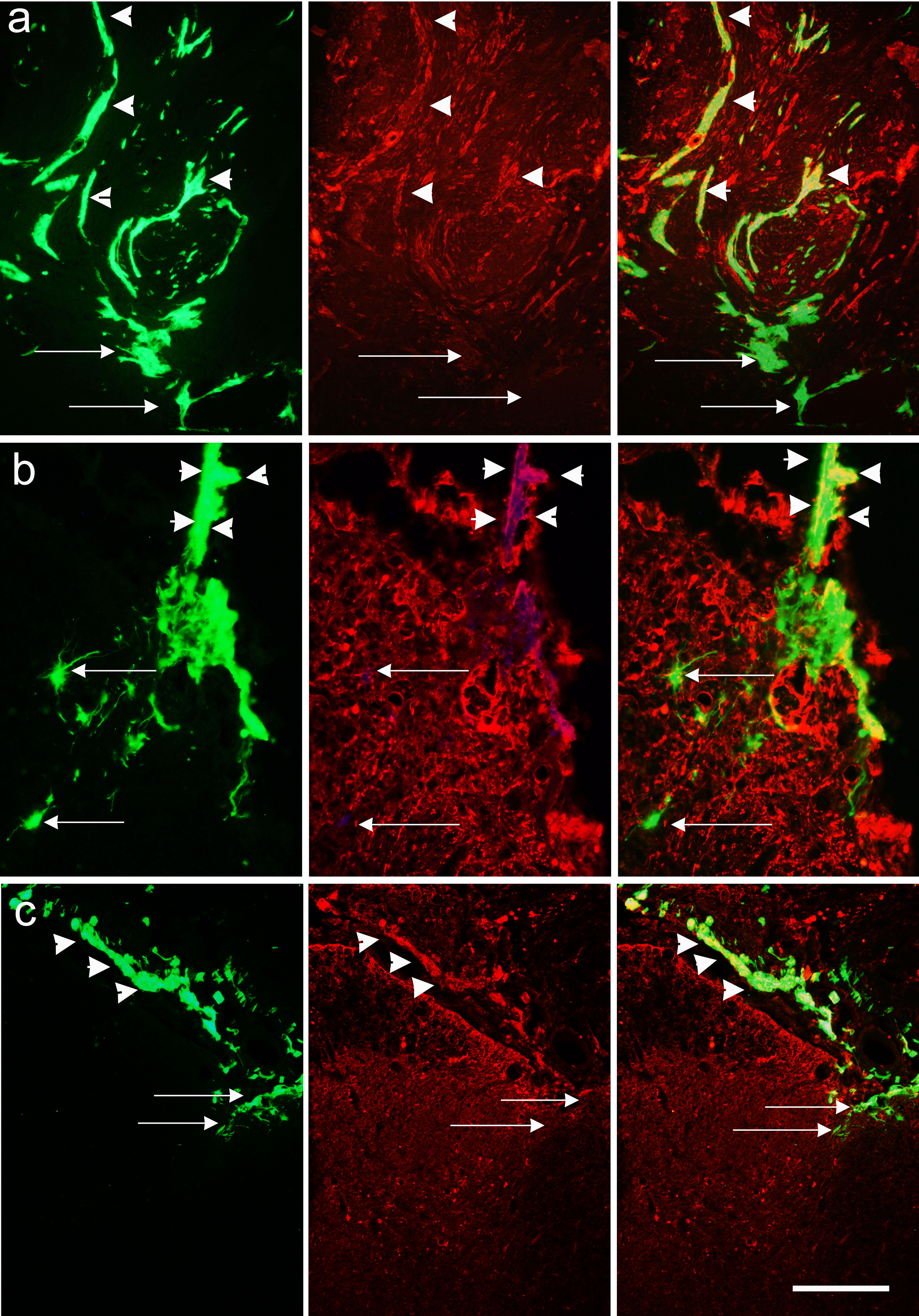

Supplement: Additional file 6: Figure S5 — bNCSCs (green) within the spinal cord are closely associated with VGlut2, a marker of glutamatergic terminals (red, arrows; blue - Hoechst). Scale bar = 10 μm. [file 1471-2202-15-60-S6.tiff]
